# Reliability and validity of an innovative high performing healthcare system assessment tool

**DOI:** 10.1186/s12913-022-08852-z

**Published:** 2023-03-13

**Authors:** Anwer Aqil, Kelly Saldana, Naeem uddin Mian, Mary Ndu

**Affiliations:** 1 Credence Management Solution, LLC, GHTASC, Institutional contractor USAID, Senior HSS MEL Advisor, Office of Health System, USAID, Washington, D.C., USA; 2 Systems Strengthening and Resilience, Abt Associates, Rockville, USA; 3 Contech International, Lahore, Pakistan; 4 Health and Rehabilitation Sciences, University of Western Ontario, London, Canada

**Keywords:** Universal health coverage, Health systems, Performance, Accountability, Accessibility, Affordability, Reliability, Validity, System practice

## Abstract

**Background:**

Universal Health coverage (UHC) is the mantra of the twenty-first century yet knowing when it has been achieved or how to best influence its progression remains elusive. An innovative framework for High Performing Healthcare (HPHC) attempts to address these issues. It focuses on measuring four constructs of Accountable, Affordable, Accessible, and Reliable (AAAR) healthcare that contribute to better health outcomes and impact. The HPHC tool collects information on the perceived functionality of health system processes and provides real-time data analysis on the AAAR constructs, and on processes for health system resilience, responsiveness, and quality, that include roles of community, private sector, as well as both demand, and supply factors affecting health system performance. The tool attempts to capture the multidimensionality of UHC measurement and evidence that links health system strengthening activities to outcomes. This paper provides evidence on the reliability and validity of the tool.

**Methods:**

Internet survey with non-probability sampling was used for testing reliability and validity of the HPHC tool. The volunteers were recruited using international networks and listservs. Two hundred and thirteen people from public, private, civil society and international organizations volunteered from 35 low-and-middle-income countries. Analyses involved testing reliability and validity and validation from other international sources of information as well as applicability in different setting and contexts.

**Results:**

The HPHC tool’s AAAR constructs, and their sub-domains showed high internal consistency (Cronbach alpha >.80) and construct validity. The tool scores normal distribution displayed variations among respondents. In addition, the tool demonstrated its precision and relevance in different contexts/countries. The triangulation of HPHC findings with other international data sources further confirmed the tool’s validity.

**Conclusions:**

Besides being reliable and valid**,** the HPHC tool adds value to the state of health system measurement by focusing on linkages between AAAR processes and health outcomes. It ensures that health system stakeholders take responsibility and are accountable for better system performance, and the community is empowered to participate in decision-making process. The HPHC tool collects and analyzes data in real time with minimum costs, supports monitoring, and promotes adaptive management, policy, and program development for better health outcomes.

**Supplementary Information:**

The online version contains supplementary material available at 10.1186/s12913-022-08852-z.

## Background

Emerging threats from infectious disease, ongoing demographic shifts characterized by continued growth of both young and ageing populations and urbanization, climate change and its associated extreme weather aftermath, as well as increasing social and economic disparities, reemphasize the importance of strengthening health systems to meet the health consequences of these challenges. The World Health Report [1] on improving health systems brought attention to the health system as vehicle to improve health of the people and the need to make health systems improvements a donor priority. Subsequent donors’ focus on health systems strengthening along with the development of multiple heath system strengthening (HSS) frameworks [2–16], have contributed to a better understanding of health system actors (public, private, community and outside of health system), functions, resources, services, and outcomes. However, development of the evidence base has not paid sufficient attention to the interactions among system components. The result is that evidence related to the impact of HSS interventions is often difficult to replicate and correlation to health outcomes are weak, leading some to question the overall effectiveness of HSS interventions and investments.

Limited investments in HSS not only weaken evidence generation but also have repercussion on achieving the United Nations [17] Sustainable Development Goal (SGD-3) target (3.8) to “Achieve universal health coverage (UHC), including financial risk protection, access to quality essential health-care services and access to safe, effective, quality, and affordable essential medicines and vaccines for all”. The measurement of UHC through SDG indicators 3.8.1 and 3.8.2 includes essential services coverage and financial hardship [18]. Tracking progress towards UHC using these outcomes indicators is important. However, UHC is a multidimensional concept that is reflective of a system’s capabilities, society’s cultural values [19], priorities and power relationships [20, 21], and therefore requires more nuanced diagnostics to enable programmatic response to the measurement. For example, access is more than simply the availability of services, they must also be affordable, and people must have confidence in their effectiveness [22]. Accountability is required to ensure this confidence and to guard against corruptive influences; to incentivize consistent (and improved) performance overtime [23–33]. Reliable care processes also underpin access and performance as they are critical to ensuring client use of the system. Achievement of UHC is grounded in social values like solidarity, equity, efficiency; it also involves contributions of individuals and communities in taking care of their own health. The challenge in tracking UHC progress is how to incorporate the processes that underscore the dynamic relationship between social values and the unique roles of systems actors in a measurement tool that also serves to help assess healthcare system performance**.**

The purpose of this paper is to describe a HPHC conceptual framework and tool to address the need for assessing essential processes that lead to AAAR care and their linkages to health system performance outcomes including UHC; creating testable hypotheses for measuring changes in a health system that are reflective of HSS interventions, bridge the evidence base between HSS and UHC, and can support increases to domestic and donors’ investment in HSS. This paper will answer the research question, “is the HPHC tool reliable and valid?”

The paper is organized in different sections starting with a literature review around HPHC AAAR constructs followed by methods and results on reliability and validity of the tool. In the discussion section, the paper comments on the tool’s added value, complementarity to other HSS tools and on how the tool could be used to improve the performance of a health system including its ability to achieve health outcomes and UHC. Lastly limitations and conclusions are presented.

## Conceptual framework

**Fig. 1:**
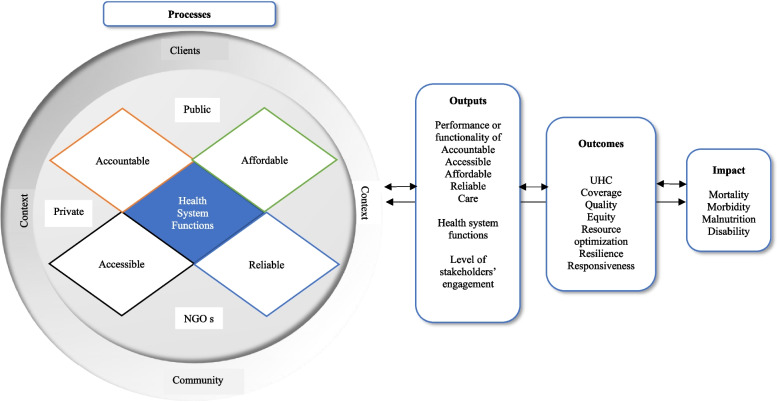
High performing health care conceptual framework

The United States Agency for International Development (USAID) developed the HPHC framework [34] to further a collective understanding of the characteristics of strong health systems. By articulating a set of characteristics across four AAAR dimensions, the framework seeks to describe the role those different stakeholders across society play in a strong health system and to demonstrate that both supply and demand side actions contribute to a health system’s functionality. The HPHC framework presumes that to deliver needed health care reliably to clients/communities in ways they trust, and can afford, requires that the people/communities have a voice in shaping that care, and therefore, that healthcare needs to be simultaneously accountable, affordable, accessible, and reliable (AAAR; Fig. 1), irrespective of whether it is delivered through the public or private sector/non-government organizations (NGOs).

The HPHC framework postulates that the AAAR dimensions of healthcare must be present and functional to truly achieve UHC because addressing processes of care is the responsibility of everyone [10]. The framework recognizes that a health system requires collective action by multiple actors from national to local levels and across community, public and private entities (Fig. 1). The accountable and reliable attributes of healthcare not only make the institutions included in the health system as the owners and organizers of health care, but also make them collectively responsible for assuring health care is available when people need it and can afford it without undue burden. Therefore, despite being distinct constructs, AAAR are interrelated. The HPHC framework acknowledges a well-functioning health system’s dual role as both supplier and demand generator for health services. The framework also recognizes the community’s critical and dynamic relationship with health system as consumer, advocate, overseer, and overall partner in ensuring that community health needs are met and UHC is achieved. The framework identifies under AAAR domains, the major salient health system processes, six building blocks/functions, stakeholders, and context comprehensively and acknowledges their interrelationships (Fig. 1), which typically have been limited in other HSS/UHC measurements related to outcomes.

Measuring the functionality of system processes/building blocks adds value to this space because these processes are critical to improve health system performance, outcomes, and impact (Fig. 1). The relationships among processes, outputs, outcomes, and impact are not linear in the framework, but dynamic; they interact and affect one another to drive toward systemwide changes (two-ways arrow). Achieving better UHC outcomes/impact requires HSS interventions that improve the weak or non-functional system processes. As specific processes get improved other weakness may be exposed or become more important thus, frequent measurement of such processes complemented by progress at the outcome level can create a cycle of continuous improvement. Measurement of the perceived functionality of processes when combined with data from other sources on health system performance provide richer insights into where additional action might drive better performance.

The HPHC framework and its tool are grounded in theories of system practice, process/quality improvement [35, 36] (to holistically oversee, reform, and measure system components/processes interactions and impact), organizational development [37–39] (to manage organizational change and learning), behavioral sciences [40–43] (to change practices and social norms for better performance), and empowerment [44–46] (for greater clients’ autonomy and community’ voice in shaping health systems).

The HPHC framework’s emphasis on effective processes adds value by: identifying major HPHC processes and their functionality; situating the locus of control for change within the health system (broadly defined to reflect institutions across society); empowering communities and fostering a dynamic relationship for joint actions and mutual accountability for better health outcomes; creating opportunities for continuous improvement as systems work toward these ideals; enabling future evidence generation related to testing hypotheses about AAAR and their sub-attributes’ relative contribution to health system outcomes; supporting innovative ways for designing, managing, monitoring, and evaluating health system strengthening and contributing to the state of the art knowledge.

### HPHC tool

This section provides further elaboration of literature on the AAAR dimensions of the HPHC framework, and their operational definitions and boundaries. The section next addresses the rationale of HPHC tool using perceptions, and how it counters potential biases. Lastly, this section describes different modalities to use the tool.

### AAAR literature and operational definitions

The HPHC tool is based on the “Access to Universal Health Coverage through High Performing Health Care” fact sheet [34] which provided operational definition of AAAR and its subdomains (Table 1). These operational definitions were used to select five indicators that were important and relevant to measure the specific AAAR sub-domains. Further details on selected indicators for subdomains are provided in the face and content validity section. The complete tool is available upon request (Additional file 1).

**Table 1:** HPHC AAAR domains and subdomains by Cronbach Alpha (overall alpha = 0.92); *N* = 213

In Accountable Healthcare system Alpha = .93	In Affordable Healthcare system Alpha = .80	In Accessible Healthcare system Alpha = .95	In Reliable Healthcare system Alpha = .96
Account_1 ● Communities, civil society, and the private sector engage with local, regional, and national governments as partners in the management, funding, and oversight of health institutions. **Alpha = .81**	Afford_1 - ● Routine or unexpected health care costs, including medications or supplies, do not impoverish people. **Alpha = .58**	Access_1: ● Health facilities and medicines are located within a reasonable distance and are consistently open on a regular schedule known to the community and have the staff and equipment to fulfill their designated functions. **Alpha = .82**	Reliable_1 ● Health facilities and health workers have the right supplies and quantity of commodities needed to deliver care, including running water and sanitation services, reliable sources of energy, and appropriate procedures to prevent infections. **Alpha = .75**
Account_2 - ● Mechanisms are in place to ensure patients’ privacy and satisfaction with care. **Alpha = .74**	Afford_2 - ● People continue to seek needed care after considering the total cost of that care (e.g., the cost of services, drugs, supplies, transport, and care for family members left behind or who accompany the patient). **Alpha = 88**	Access_2: ● Alternative care options exist to extend the reach of traditional health facilities, including both paid and voluntary community health workers, as well as digital or e-health applications, drug shops/pharmacies, mobile outreach, etc. **Alpha = .80**	Reliable_2 ● Health workers have the knowledge, skills, motivation, credentials, and cultural understanding to provide care; are engaged in continuing education; are regulated through professional and licensing associations and supported through retention strategies. **Alpha = .86**
Account_3 -● Information regarding health financing, delivery, and outcomes (at a population level) are publicly available. **Alpha = .79**	Afford_3 ● People opt to participate in pre-payment schemes or insurance plans to improve their ability to access health care and protect themselves from financial hardship because of illness. **Alpha = .73**	Access_3 -● Quality of care for priority interventions meets established standards from both providers and consumers, and is consistent across all facilities, regardless of whether they are public, private, non-profit, or faith-based. **Alpha = .89**	Reliable_3 ● Health facilities are able to meet the standards of accrediting organizations, and effectively engage their communities, and the people they serve. **Alpha = .84**
Account_4 ● Good health outcomes, supported by self-directed and family-centered positive health behaviors, are sustained. **Alpha = .68**	Afford_4 -● Governments allocate financial and human resources for health to meet priority needs, and work with the private sector and civil society to increase domestic funding and ensure the adequate distribution of such resources **Alpha = .58**	Access_4 -● Emergency health care and related transportation are available. **Alpha = .81**	Reliable_4 ● Pharmaceutical and logistics management systems are in place so that medicines, devices, and commodities are safe and of expected quality, and with controls that minimize the risks of theft or falsification. **Alpha = .90**
Account_5 ● Licensing agencies and professional organizations are responsible for credentialing providers, accrediting facilities, and setting standards in partnership with national (and/or local) government. **Alpha = .74**	Afford_5 - ● Safe and effective essential medicines are available without undue financial hardship to obtain them, along with mechanisms to ensure their responsible use (at the right time, for the right conditions, and at the right doses). **Alpha = .78**	Access_5 -● People understand when, why, and where to get the care they need and are motivated to seek it. **Alpha = .88**	Reliable_5 ● Health workers are safe from violence, assault, or harassment and are protected against natural disasters, disease outbreaks and emergencies. **Alpha = .87**
Account_6 -● Recourse or appeal options are available for patients or communities dissatisfied with health care. **Alpha = .78**		Access_6 -● Providers deliver health care in a manner that ensures equitable health outcomes and promotes dignity and respect for all patients and providers. **Alpha = .87**	Reliable_6 ● Patients trust that institutions and providers will give them the care they need in a way that meets their needs respectfully, without stigma, shame, fear, or abuse. **Alpha = .87**
			Reliable_7 ● Care continues during times of disruption, shock, or crisis. **Alpha = .90**

### Accountable healthcare

Murray [13] defined health system responsiveness as meeting the non-medical needs of the population and as an outcome of the governance function. Accountability to the target population is implicit in responsiveness construct but never explicitly stated or measured. Lewis [47] has drawn attention to linkages between minimal funding levels, illegal payments in publicly financed and delivered care and limited accountability. Peters [48] noted that accountability measures in health systems, as a part of regulatory or oversight efforts, are relatively neglected in comparison to organization and financing of services. Cleary et al. [21] building on the concepts of bureaucratic (internal) and external accountability [49] showed that there is a gap in the power relationships among mechanisms of accountability. The relatively powerful bureaucracy with an emphasis on supervision and management systems and focused on compliance to centrally defined outputs and targets can constrain front line managers and providers from responding to patient and population priorities. Thus, accountability is best understood as refereeing the dynamics in two-way relationships, often between unequal partners [20].

Recognition of this unequal partnership became the rally point for the social accountability movement. This movement made important contributions by emphasizing citizens’ demand for and role in developing accountability processes such as assessment, demand articulation, feedback and negotiation with providers, changes in providers’ behavior and facility practices [20, 50]. Social accountability also contributed to accountability measurement through development of social audit, community score card and Citizen Charter [51–53]. Informal social accountability [54] in the context of community-based workers also highlights the social pressures borne by health workers and how informal social networks create incentives/disincentives for health worker accountability and relationships to formal accountability structures. A robust need for data for accountability is undeniable but evidence of the weakness of information systems in low-and-middle-income countries (LMIC) is well documented [55].

Building on existing literature and recognizing that progress toward the achievement of UHC requires explicit accountability structures within country health systems. Under HPHC framework, accountable health care means society as a whole works together to ensure care meets people’s needs, which brings in the WHO [10] concept that the health system is everyone’s responsibility. In the HPHC framework ‘everyone” is implied by the system’s stakeholders (clients, communities, public, private, NGOs, donors, international organizations); and each of their roles are considered in promoting accountability. Accountability is measured through six subdomains (Table 1) comprising of Public-private/civil society organizations (CSO) partnership for management, funding, and oversights of health institutions; patient privacy and satisfaction, transparency in information sharing on financing, service outcomes, sustaining clients’ health behaviors and outcomes, regulatory processes for quality, and recourse options available to dissatisfied clients. An overall accountable score provides level where health system stands in providing Accountable care. The Accountable sub-domains scores highlight strengths and weaknesses under each sub-domain.

### Affordable healthcare

Adequate financing of the health system across public, private and Non-Government Organization (NGO) sectors, to make healthcare affordable has remained problematic. Stenberg et al. [56] estimated an additional $274 billion global spending on health is needed per year by 2026–30 to make progress towards the SDG3 targets where health system absorptive capacity is constrained and US$371 billion would be needed by 2026–30 to achieve SDG3, necessitating more revenue generation and multisectoral collaboration for addressing social determinants of health. However, revenue generation needs to be fair and progressive to support the overall affordability of the health system because, for example, simply instituting user fees would in turn affect access to care [57–59]. A range of financial barriers affect access to healthcare, and the financial health burden of accessing care is a major cause of household’s impoverishment. Financial protection schemes such as health insurance [60], cash transfer [61], public health expenditures [62] have been shown to reduce out of pocket expenditure and improve overall affordability. Affordability is also affected by the cost of medicine, laboratory tests, x-ray, and the costs to individuals and families of seeking care such as cost of transport or childcare. Additionally, there is an economic cost to both individuals and populations of substandard and falsified medicines and [63–65] substandard medicines have been noted as a contributing cause of increased inequity in health outcomes in Uganda [66].

Under HPHC framework, affordable health care means money spent on care provides the best value possible. It is assessed through five sub-domains (Table 1) reflecting that costs do not impoverish people, that people consider the total costs before seeking care, and that availability and use of pre-payment schemes help to protect from financial hardship. Affordability also requires governments to allocate resources effectively and build public-private partnerships to increase domestic funding and ensure availability and rational use of safe and quality medicines without financial burden. A summary affordable score provides information on the overall level of affordability of care in and within the health system. The affordable sub-domains scores identify strengths and challenges under each sub-domain.

### Accessible healthcare

Tanahashi [67] provided a pathway for access to care that started with availability of health services, geographic and financial access, followed by a need for acceptability and actual receipt of care while adding a final step of effective coverage, meaning that care is of such quality that it has produced the intended positive effects. Thus, he created a linkage among access (those who can use the service), coverage (those who utilize the service), and quality to make effective service coverage (=quality*coverage). Perceptions of quality also affect access to care [68]. Acceptability broadens the concept of access to include other sociocultural factors affecting access to health care such as gender norms, respectful care, and empowerment [69–71]. Thus, access to health care is a multidimensional, and complex social construct [22]. It reflects not only how many people have access to services but also reveals inequities in access within social and vulnerable groups/communities, and within and between countries.

Accessible healthcare, per the HPHC framework exists when care is available when and where people need it and can use it and meets quality standards. It has six subdomains (Table 1) that cover geographical access to facility, staff and medicine, alternative options including eHealth to extend reach of care, emergency care, quality, cultural access through respectful care and achievement of equitable outcomes. An overall accessible score provides level of access to health care. The accessible sub-domains scores highlight strengths and gaps in dealing with different access issues.

### Reliable healthcare

In health literature, reliable care is mostly treated as an aspirational goal, a dimension of quality but not as a system characteristic. However, it is a distinct element of HPHC framework, which is not featured in other health system frameworks. Reliability includes emergent concepts of resilience, which has gained increasing relevance due to COVID-19 pandemic. Dependable quality and safety remain a concern globally causing human suffering and of waste of resources [72]. High Reliable Organization (HRO) theory and practice [73–75] provides context and justification for use of this construct and has made inroads in healthcare literature [76]) with respect to systems processes, interdependencies among units and levels, and to create a high degree of accountability. To be a reliable organization [77] requires preoccupation with failure, reluctance to simplify interpretations, sensitivity to operations, commitment to resilience and deference to expertise in time of crisis, which implies a focus on interdependence, redundancy, and culture [78]. The Joint Commission [79] in United States of America (USA) has made creating and maintaining a culture of safety a requirement for the healthcare organizations accreditation [80]. The greater use of reliability-enhancing work practices such as respectful interaction and mindful organizing processes were associated with better performance (fewer medication errors and patient falls) compared to organizational citizenship behavior [81] while failure to convert periodic mindful practices as habit lead to unreliable healthcare [82].

HPHC framework incorporates the latest insights from the existing literature on reliable care. It defines Reliable health care as high-quality care, delivered in a timely manner that promotes dignity and respect for all patients and providers. This operational definition has seven characteristics (Table 1). Reliable health care assumes not only client safety through error free care but includes a time dimension that such care is replicable and consistent. Reliable care also entails resilience that in times crisis processes are inbuilt to continue providing care. An overall reliable score provides information on to what extent health care is reliable. The reliable sub-domains scores highlight strengths and weaknesses under each sub-domain.

#### HPHC tool boundaries

HPHC tool is confined to measuring functionality of AAAR processes for multiple reasons: to create a holistic picture of major processes that not only reflect direct processes of care but include the health system functions/building blocks, engagement of diverse stakeholders such as clients/communities, public/private/NGOs organizations and other multi-sector factors where changes can be made to affect health system outcomes/UHC and performance; to avoid participants’ fatigue with a long questionnaire; and to foster enhanced use of the existing sources of information that gather health system outcomes data, especially the routine information system. AAAR functionality scores should be combined with health system performance and outcomes data for understanding the linkages. Non-alignment of perceptions about functionality of healthcare processes with measures of performance through routine or survey data can provide information on where to focus efforts for improvement and the development of effective interventions. Building on the quality/performance improvement literature, outcome is the byproduct of effective processes, therefore, the tool creates opportunities to test the hypothesis that higher functionality of AAAR processes is associated with better outcomes.

#### Use of perceptions

The tool assesses the functionality of AAAR processes through individuals’ perceptions because people base their actions on their perceptions, impressions, and views [83]. Objective reality does not always reflect actual practice, for example, policy and regulatory standards might be available on paper but actual practice might differ [84, 85]. Perceived health status is predictive of mortality and its association with objective health status is well documented [86–88]. These illustrated examples show that perceptions are a good proxy of objective reality. Therefore, we have used perceptions to assess level of overall performance and whether care is accountable, accessible, affordable, and reliable. The tool accounts for potential perception bias by including anonymity, instructions to be truthful and adding respondents’ characteristics to check and control for biases during analyses [89–92].

#### Modality of use

HPHC is a web-based tool (https://hphctool.org), which can be shared with many people in different locations. The tool improves representation and solves issues of cost and frequency of data collection, facilitating monitoring progress at low cost. In addition, HPHC can be applied through different modalities such as key informants, or the Delphi technique if all stakeholders are represented. The tool could be used in a survey using small sample size lot quality assurance sampling (LQAS) for overall estimate of HPHC scores. LQAS could also be used to assess the country/region/district is meeting HPHC predetermined level of performance.

## Methods

### Data collection instrument

The HPHC tool has 120 questions items spread under AAAR subdomains.

### Sample size for reliability and validity

The sample size of 200 is calculated based on literature recommendations for testing reliability and validity considering how many respondents needed based on number of questions in the tool [93–98]. Sample size calculation for reliability accounts for expected Cronbach alpha = 0.75, type 1 error = 0.05, number of items = 120, and power = 80; and alpha = .8 requires lower sample size.

#### Survey design

The survey design was cross-sectional.

#### Method of data collection

We used internet survey because it is cost-efficient and easier to reach eligible participants in LMIC.

#### Eligible participants

All those age 18 years or more, who interacted with health system as part of the health system in some capacity or a client were eligible to participate in the survey to have broader representation from in and outside the system. It is assumed that if the health system has strong linkages with clients/communities, clients will be able to expresses their opinions about health system processes functionality. The survey was only available in English and thus required respondents to have English language skills. No personal information was collected except information about gender, age, types of organizations, and years of work experience. The research was conducted in compliance with the United States Federal Codes of Regulations (CFR) on protection of human subjects, including informed consent (22 CFR 225). The research protocol and ethical statement were approved by Kelly Saldana, Director, Office of Health Systems, USAID, who has the authority and determined that the research protocol met the criteria for exemptions from the review by Institutional Review Board committee (22 CFR 225.104).

#### Sampling

We used non-probability sampling to recruit volunteers from LMIC using different global networks (Health System Strengthening Network, Routine Health Information system network, Christian Connections for International Health hospital network) and a consulting firm in Pakistan to reach public, private, NGOs, and international organizations in health/development sector and people with different socio-demographic characteristics such as age, education and employment for broader representations. Given no access to the networks’ memberships, it was not possible to randomly select specific number of participants from different countries. The use of non-probability sampling remains the dominant method for testing reliability and validity of new constructs for the last 40 years [99–101].

Convenience sampling is a dominant method for collecting reliable and valid information in development field such as World Bank Governance indicators [102] and Country Policy and Institutional Assessment tool [103] or Transparency International Corruption Perception Index [32]. The results of non-probability sampling are comparable to probability sampling when all stakeholders in the target populations are represented, and respondents’ socio-demographic characteristics do not influence the responses [104–108]. We addressed representation in non-probability sampling by involving health and development professionals in multiple countries and to account for survey participants’ socio-demographic characteristics and types of organizations (public, private, non-government, and international organizations) influence on their perceptions of AAAR processes functionality.

#### Types of analyses

Out of 219 respondents [from 37 countries in **Africa** (Cameroon, Côte D′ Ivoire, Ethiopia, Ghana, Guinea, Kenya, Lesotho, Madagascar, Malawi, Mali, Nigeria, Senegal, Sierra Leone, South Africa, South Sudan, Tanzania, Uganda, Zambia, Zimbabwe); **Asia** (Bangladesh, India, Indonesia, Jordan, Kazakhstan, Myanmar, Nepal, Pakistan, Philippines, Uzbekistan, Yemen); **Latin America/Caribbean** (Antigua & Barbuda, Colombia, Guyana, Haiti, Peru)], we excluded six respondents from USA and Canada from analysis because we wanted to test the tool in LMIC. To contextualize where these countries stand on their health system performance, we have used the universal health coverage index (UHCI) [109] for comparison. UHCI is a good proxy for health system performance because it is comprised of service coverage and other indicators. The overall participants’ countries UHCI ranges from 28% (Madagascar) to 77% (Peru). However, in most of the African and Asian countries, UHCI is below 50%, indicating that these countries have a long way to achieve UHC.

Social desirability was tested by assessing whether the responses to negatively worded statements were in alignment with positive statements to show same direction of responses as well as spread of the responses. Cronbach’s Alpha was used to test the internal consistency of the overall tools and its four domains. Construct validity was tested using correlations among the four AAAR construct. Lastly, validation was conducted by comparing tool results with similar data from other sources of information.

## Results

The results are based on 213 people from 35 countries in Africa, Asia, and Latin America. Pakistan, Nigeria, India, and Philippines had 20 or more people respondents making it possible to answer whether the tool could collect reliable and valid information in different contexts/settings. Thus, the results are presented overall and by these selected four countries.

### Respondents’ socio-demographic characteristics

Overall, 67% respondents were males and countries with 20 or more respondents showed similar distribution except for Nigeria where slightly more women responded (Table 2). The respondents were almost equally distributed among public sector, private sector/NGOs, and international organizations. However, in Pakistan more respondents were working in public sector, while in Nigeria and Philippines the dominant category was private sector and majority respondents in India were from NGOs. The respondents overall and in selected countries had more than 15 years of education and professional experience. There were no significant differences between overall HPHC score and: a) types of organizations (SS = 557, df = 3, F = 1.52, *p* = .211), b) respondents’ experience (SS = 129, df = 3, F = .35, *p* = .79), c) education (SS = 357, df = 2, F = 1.45, *p* = .23), and gender (SS = 258, df = 1, F = .211, *p* = .14) using ANalysis of VAriance (ANOVA) test, indicating that these characteristics did not influence the HPHC scores.

**Table 2:** Percentage distribution of respondents’ characteristics by all and selected countries

Respondents’ characteristics	All (*N* = 212)	Pakistan (*N* = 93)	Nigeria (*N* = 28)	Philippines (*N* = 21)	India (*N* = 21)
Gender					
Male	67.6	76.4	64.3	76.2	71.4
Female	32.4	23.6	35.7	23.8	28.6
Types of Organization					
Private Sector	11.4	21.5	3.6	4.8	0
Civil society/NGOs	22.4	13.9	21.4	23.8	61.9
Public Sector	33.3	52.6	17.9	23.8	9.5
International organizations	32.9	11.8	57.1	47.6	28.6
Professional Experience					
< 10 years	23.3	28	17.8	4.8	33.3
10–14 years	24.8	22.6	39.3	23.8	23.8
15 years or more	51.9	49.4	42.9	71.4	42.8
Education					
< 10 years	7.6	7.5	7.1		14.3
10–14 years	13.4	9.7	28.6	14.3	9.5
15 years or more	79	82.8	64.3	85.7	76.2

### Social desirability bias test

**Fig. 2:**
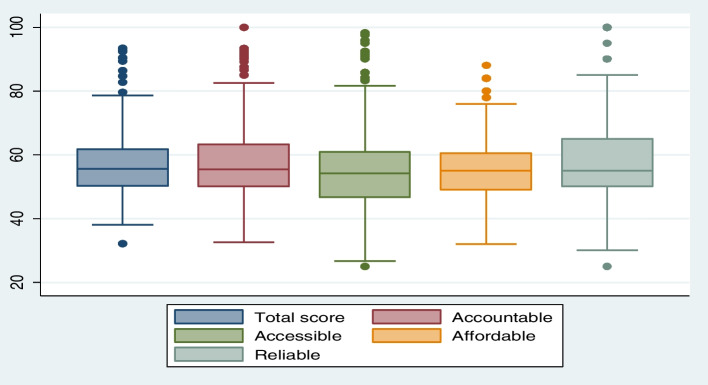
Percentage distributions of HPHC total scores and by AAAR dimensions

**Fig. 3:**
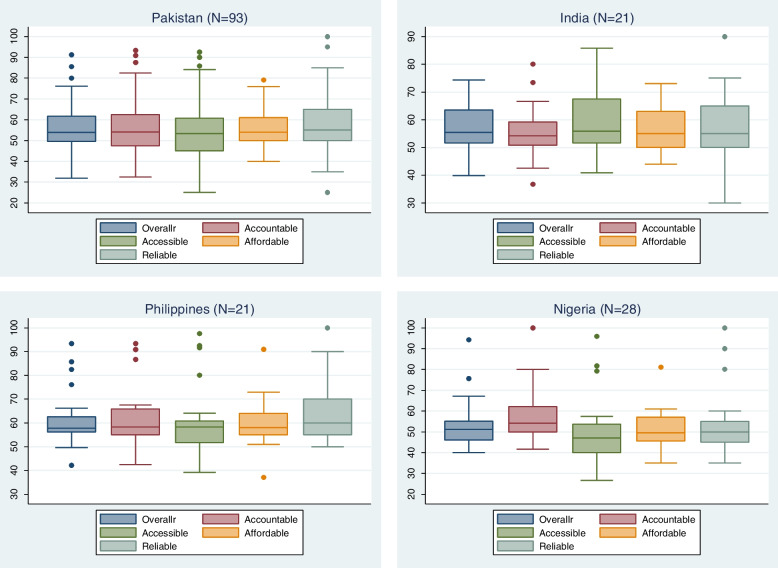
Percentage distributions of HPHC total scores and by AAAR dimensions by selected countries

The analysis showed that respondents’ rating on positive and negatively worded statements were aligned showing same direction and no social desirability bias. Descriptive analyses of data showed wide spread of overall HPHC scores and its domains (Fig. 2) indicating that the respondents were expressing their opinions freely without any social desirability bias, which if present could have titled score in positive direction. This trend was also observed in the selected countries data (Fig. 3). The respondents’ characteristics were not found to be associated with overall high performing and AAAR domains scores, indicating their no contributions affecting responses.

### Reliability of the tool

Reliability of the tool is tested in two ways. Through internal consistency test and whether the internal consistency is repeatable in different contexts/settings.

### Internal consistency

The tool has an overall Cronbach Alpha of .92, indicating that the items and responses were internally consistent, showing reliability. Similarly, the major domains; accountable, accessible, and reliable sub-scales have alpha above .9 and for affordability 0.8 (Table 1). It also showed alpha for all four subdomains. Each subdomain has five items (Additional file 1. tool questionnaire) and have high internal consistency. Nunnally [110, 111] recommend that low reliability coefficients 0.5–0.6 are tolerable when the exploration of constructs is at the initial stage of construct exploration, otherwise alpha of 0.7 or more is a reliability standard of acceptability. The Table 1 shows that reliability standard was met by almost all subdomains except two affordability subdomains. However, these coefficients are in acceptable range given the initial stage of tool test.

### Reliability in different contexts

We also tested repeatability of the alpha in Pakistan, Nigeria, Philippines, and India that met the sample size requirement of 9 or more respondents for scale 25–35 items for testing alpha at .8 or higher level [112–114]. We found an alpha >.80 in all four countries, which reflects that the tool reliability is repeatable in different countries and settings.

### Validation of the tool

The tool was validated testing face, content, and construct validity, and confirming validity in different contexts and through triangulating tools findings from other sources of information.

### Face and content validity

Face and content validity was checked through four steps. First, the HPHC tool was shared with 15 public health experts to check face and content validity and add/delete/revise question statements related to AAAR subdomains. Second, the tool was circulated within lead authors’ organization to broaden assessment of face and content validity and to solicit suggestions for improvement. Third, the revised tool was circulated to field staff of the lead authors’ organization with expertise in public health around the world for their review and comments. The country field staff confirmed tool items appropriateness, specificity, and relevance. Fourth, the final edited tool was again shared with a select group of public health advisors who concurred with earlier respondents group findings of tool face and content validity.

### Tool construct validation

The HPHC framework hypothesizes that its four domain constructs: accountable, accessible, affordable, and reliable are interrelated. A correlation test among these constructs (Table 3) confirmed significant associations, showing construct validity. The associations among the four constructs were also found in the Pakistan, Nigeria, Philippines, and India data indicating that the tool is also valid in different contexts and settings for measuring HPHC AAAR domains. The interdependence among the health system processes/functions under AAAR domains and subdomains makes factor and principal component analyses irrelevant for discriminant and convergent validity. The new network model approaches [115–117] where characteristics are distinct but influence each other to contribute to underlying constructs are more appropriate and promising for future analyses.

**Table 3:** Associations among the HPHC AAAR domains (*N* = 213)

	Accountable	Accessible	Affordable	Reliable
Accountable	1.0000			
Accessible	0.7786*	1.0000		
Affordable	0.6021*	0.6754*	1.0000	
Reliable	0.7178*	0.8372*	0.6960*	1.0000

*p = <.001

### Validation of tool findings from other sources

**Fig. 4:**
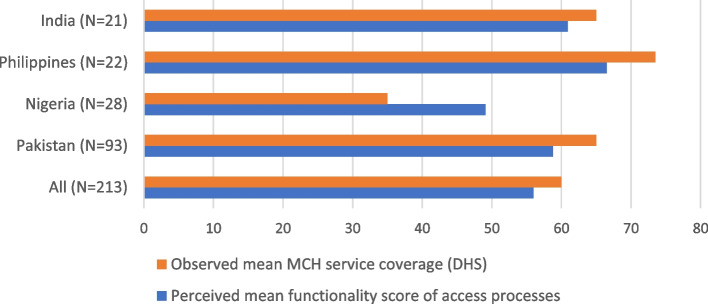
Comparison of perceived mean access processes functionality scores with WHO MCH coverage data

Triangulation of information from different sources is another method of validation of collected information. HPHC tool is unique and therefore, the overall findings are not comparable to other HS assessment tools. However, there are many indicators within AAAR subdomains that are close approximation to data collected by other sources and can be used for partial validation of HPHC tool findings. We have used this comparison with other sources of data and three examples are presented. The Fig. 4 shows that the mean perceived functionality of health facility which has staff and medicines and is a proxy for access is closely matched with mean MCH coverage estimated through demographic and health survey (DHS) [118], indicating that level of perceived functionality of access processes is comparable to actual services coverage, validating collected information.

**Fig. 5:**
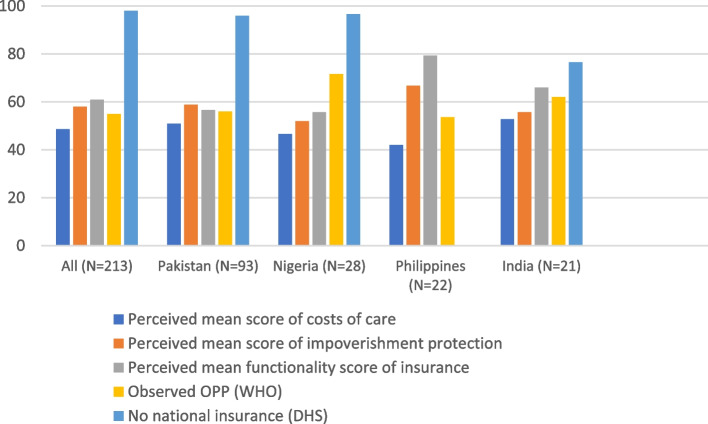
Comparison of perceived affordability processes functionality scores with external data on OPP, insurance

The perceived functionality of cost containment processes (assessed by costs of care, impoverishment protection and opting for insurance) is compared with out-of-pocket expenditure (OPP), Fig. 5, available through WHO data repository [119]. This finding validates that 50% perceived functionality of the cost containment processes is in alignment with high OPP as reported by WHO data. Limited functionality of cost containment processes is further supported by absence of national insurance schemes in 98% of the countries captured in DHS data [120].

**Fig. 6:**
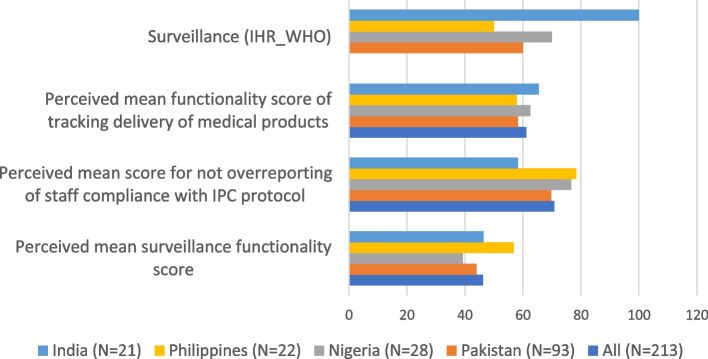
Comparison of perceived mean surveillance functionality scores with WHO IHR surveillance capacity score

Another example of validating the tool’s findings was done by comparing mean perceived surveillance functionality score with WHO International Health Regulation surveillance capacity score [120]. There is close correspondence between perceived functionality of surveillance and IHR capacity score in selected countries (Fig. 6) validating that the tool is comparable in assessing functionality of surveillance processes.

### Tool responsiveness and precision

Responsiveness of the tool addresses whether the tool could identify change over time in the respondents and in different contexts, while precision is about identifying changes in the phenomenon of interest [121]. The assessment showed that the tool was able to identify variations in HPHC scores and its domains in selected four countries (Figs. 2 and 3) showing its responsiveness and precision and validating the tool utility in different contexts. We also tested whether tool was able to find differences in overall HPHC and AAAR domains scores among selected countries (Table 4), which confirmed the tool’s ability to detect significant differences in diverse contexts. Philippine overall HPHC score is significantly different from Pakistan, India, and Nigeria. There is no significant difference between Pakistan and India overall HPHC scores, but their overall HPHC scores significantly differ from Nigeria. Due to use of non-probability sampling and small sample sizes, HPHC mean percentile performance scores of India, Nigeria, and Philippines should not be considered reflective of country performance, whereas Pakistan’s sample size is adequate and representative of all provinces, thus HPHC mean percentile performance score is reflective of Pakistan’s health system performance.

**Table 4:** HPHC tool showing significant differences in overall HPHC and AAAR scores by countries, Tukey’s method; (*N* = 163)

	Overall HPHC score	Accountable	Accessible	Affordable	Reliable
Pakistan	56.0**	56.5*	54.3*	55.2*	55.9**
Nigeria	52.4**	57.1	49.3**	51.4**	55.1*
Philippines	63.8**	64.9*	62.8*	61*	66.2**
India	57.2	55.7	59.0	57.0*	58.6

Significant *p = .10, **p < .05

## Discussion

Besides being highly reliable and valid, the tool’s uniqueness lies in its ability to assess not only performance in processes of accountable, accessible, affordable, and reliable care but also the processes dealing with the role of system stakeholders, clients/communities, public/private/NGOs, multi-sector entities in a way that opens opportunities for action at different levels and across the sectors. The tool also adds value by collecting information through use of a technology platform and: provides real time data collection and analysis; facilitates monitoring change over time; and limits costs. The tool identifies strengths and weaknesses in health system to facilitate adaptive management, policy, program, and research development.

An important contribution and implication of the HPHC tool is that accountability is beyond an elusive concept [122–125] and is being part and parcel of everyday health system performance. In addition, affordability is not confined to financial risk protection but recognizes all the factors that contribute to the cost of care, and reliable care subsumes everyday resilience [126–128] and is not limited to sporadic crisis. Access is broadened to cultural, organizational and equity issues. By measuring functionality of illustrated health system processes regularly, HPHC framework ensures transparency for two major purposes: the policy makers, managers and organizational members develop appropriate policies, incentive processes, and take management actions to curb corruption and be accountable for health system performance and outcomes for their internal and external (community, donors) clients; communities are empowered with information to have voice in decision-making process for better health status.

Limiting the tool’s boundaries to assess level of process performance is intentional to encourage use of other existing sources of information that collect information on system outcomes, and to avoid additional costs for data collection. HPHC findings could complement and explain performance gaps identified by other sources and especially reflected in routine information systems data.

There are other complimentary health system performance assessment tools available for use. Many of these tools requires observation, record reviews, and/or interviews to collect information, and often necessitate consultant labor and stakeholders’ time to implement, implying significant costs. Due to these high costs, frequency of data collection is limited, and these tools do not work for routine monitoring purposes. The Health System Assessment (HSA) tool [129] based on the health system building blocks was developed in 2007 to assess systems strengths and weaknesses. The HSA approach synthesizes existing information—from document reviews, in- country stakeholder interviews, and site visits and provides comprehensive insights across the system or specific functions of the system. Went [130] health system rapid diagnostic tool is like HSA tool but differs with heavy emphasis on a country-led design and planning process rather than a using a prescriptive (and usually very long) list of indicators and capacity building of the partners. Primary Health Care Performance Initiative (PHCPI) [131] takes a narrower approach to health system performance assessment in LMIC by focusing on PHC and filling a performance measurement gap at this level. The tool links PHC related higher level system determinants with input, processes, and outcome indicators. The tool collects information through existing information system, conducts a survey and uses key informant interview/focus groups for missing information.

Rohrer-Herold [132] reviewed the major seven health system assessment tools, excluding PHCPI, and showed that these tools are based on prescriptive diagnostics of health system building blocks/functions. There is neither a common set of indicators nor is the selection of indicators based on theoretical or strategic frameworks. In addition, there is no concrete guidance on how to adapt the tools to different cultural/health systems contexts, despite the authors stressing the flexibility and adaptability of the tool. The review concluded that a need exists to harmonize these tools, but a political will is lacking. The reviewer’s conclusion assumes that if these indicators are harmonized and adapted, changes in performance could be measured; but this view does not account for the reasons why these tools have not been adapted to date. Further, it is critical to acknowledge that rarely do changes in health systems indicators follow a linear path, even as some aspects of the system performance may be improving, thus a different approach is warranted.

The HPHC tool brings a new measurement approach based on systems practice [8, 133]. Unlike many tools requiring outside technical experts, HPHC shifts the responsibility of measurement internally through a user-friendly tool that foster empowerment to periodically review their system’s accountable, accessible, affordable, and reliable processes of care and make necessary changes for continuous improvement in processes for better outcomes.

The tool could be used by the government, private sector, NGOs, and international organizations at national or subnational levels. The tool could be used as rapid assessment by bringing major system stakeholders and key informants with representation from public, private/NGOs and international organizations. The findings could be used for priority setting, and policy, program, and research development. Implementation of the survey using lot quality assurance sampling can provide an overall estimate of HPHC performance and disaggregation by lots (districts/regions/facilities) or to test whether certain district/regions meet predetermined (or previous) HPHC performance standards. The interpretation of findings will depend on how representative the participants are and unit of analysis. The use guidance will be provided in the web-based tool.

### Limitations

HPHC tool identifies strengths and weaknesses in functionalities of AAAR processes dealing with health system functions, clients/communities, public/private/NGOs, stakeholders at different levels, and contextual factors including multi-sectoral collaboration and assumes that they affect health system outcomes (Fig. 1). Although the results are based on non-probability sample, selection bias is minimized by inclusion of a wide representation of respondents/stakeholders in the health system from thirty-five low-and-middle-income countries. Some caution and distinction are warranted that despite the high reliability and validity of the tool, India, Nigeria, and Philippines HPHC performance scores are not reflective of country performance due to small and non-probability sample, while Pakistan’s data are reflective of country performance because they are a representation all provinces and an adequate sample size. When using the HPHC tool, it is important to pay attention to sample size, sampling, and wider representation of all stakeholders in the health system to get a reliable and valid performance estimate as well as to test the reliability and validity of tool findings with statistical analyses and triangulation with other sources of data. More research is needed to: generate empirical evidence between HPHC findings and health system outcomes; understanding magnitude of change in strengthening processes of care; and to investigate network model of assessing and validating AAAR subdomains interdependence in influencing AAAR constructs scores and outcomes. Lastly, the length of the tool needs to be reduced to avoid respondents’ fatigue.

## Conclusions

High performing healthcare tool is an important and innovative contribution to the measurement of health system performance because it identifies the level of performance within specific AAAR processes’ that contribute to system outcomes. It also fosters the system stakeholders’ ownership and responsibility for improving and designing policy, program, processes intended to improve the healthcare system’s outcomes/impact. The framework and tool will evolve over time and will help refine research, analysis, system practice, and contribute to better health system performance with more country applications.

## Supplementary Information

**Additional file 1.**

## Data Availability

The datasets generated and/or analyzed during the current study are not publicly available due to privacy and confidentiality of the respondents but are available from the corresponding author on reasonable request.

## Abbreviations

WHO: World Health Organization
HSS: Health System Strengthening
UN: United Nations
SDG: Sustainable Development Goal
UHC: Universal Health Coverage
HPHC: High Performing Health Care
AAAR: Accountable, Affordable Accessible, and Reliable
USAID: United States Agency for International Development
LMIC: Low-and-Middle-Income Countries
CSO: Civil Society Organization
NGO: Non-Government organization
HRO: High Reliable Organization
USA: United States of America
DHS: Demographic and Health Survey
OPP: Out-of-Pocket expenditure
IHR: International Health Regulation
HSA: Health System Assessment
PHCPI: Primary Health Care Performance Initiative
CFR: Code of Federal Regulations
UHCI: Universal Health Coverage Index
