# Supplementary material for: Reliability and validity of an innovative high performing healthcare system assessment tool
Source: BMC Health Serv Res. 2023 Mar 13;23:242. doi: 10.1186/s12913-022-08852-z (PMC10009863; doi:10.1186/s12913-022-08852-z)
Supplement: Supplementary file 1 — Additional file 1. [file 12913_2022_8852_MOESM1_ESM.doc]

# Appendix A: High Performing Health Care System Assessment Tool

USAID has developed [High Performing Health Care](../../../(https:/www.usaid.gov/sites/default/files/documents/1864/Access_to_UHC_through_High-Performing_Health_Care_USAID.pdf%20)) System framework and analytical tool to understand, "to what extent is the healthcare system accountable, accessible, affordable and reliable?" The information will help us identify the strengthens, weaknesses and interrelationships among these domains contributing to High Performing Healthcare system. We will use the collected information for developing recommendations for improving the system processes and performance in your country.

We would like to know your perceptions about these four domains of the healthcare systems. There are no right and wrong answers, only your opinions. Please note that your responses will remain confidential. In addition, the confidentiality will be further protected by presentation of aggregated or group data.

There are multiple statements listed under the accountable, accessible, affordable, and reliable domains. The statements are rated on the functionality scale of 0-3. Zero (0) reflects non-functional or non-existent and a three (3) represents functional all of the time. If you do not know or think that that statement is not applicable, then please use zero response category. Please select the response code which reflects your best opinion.

We are pretesting this tool, therefore, please share your comments/suggestions in the last section to improve it. This form will require 50-75 minutes to complete it. We appreciate your time and assistance. Thank you.

| **1. Country** | | **2.Province/region** | | | **3. District** | |
| --- | --- | --- | --- | --- | --- | --- |
| **4. Types of organization** | 4.1. Public sector | | 4.2. Private sector | 4.3. NGOs/CSOs | | 4.4. International organization/Donor |
| **5. Year of employment** | 5.1. Less than 5 Years | | 5.2. 5-9 years | 5.3. 10-14 year | | 5.4. 15 years or more |
| **6. Years of education** | 6.1. Less than 10 Years | | 6.2. 10-14 years | 6.3. 15 year or more | |  |
| **7. Sex** | 7.1. Male | | 7.2. Female | 7.3. Other | |  |

| **In Accountable Health care** | **Indicators**  ***Please select the response code which expresses your best opinion about the following statements. If you do not know or think that that statement is not applicable, then please use zero response category. In ACCOUNTABLE healthcare*** | | | | |
| --- | --- | --- | --- | --- | --- |
| **● Communities, civil society, and the private sector engage with local, regional, and national governments as partners in the management, funding and oversight of health institutions;** | 1- Mechanisms are in place to inform community, leaders and civil society organizations (CSOs) about health system performance, outcomes and health issues | 0.None of the time | 1. Some of the time | 2. Most of the time | 3. All of the time |
|  | 2-Mechanisms are functional to mobilize and empower communities for generating demand and advocating for health services | 0.None of the time | 1. Some of the time | 2. Most of the time | 3. All of the time |
|  | 3- Health management committees are functional at different levels of health system with members from the community, CSOs to plan, manage, and monitor health programs and performance | 0.None of the time | 1. Some of the time | 2. Most of the time | 3. All of the time |
|  | 4- Print and electronic media are major partners in overseeing management and outcomes of public/private health institutions | 0.None of the time | 1. Some of the time | 2. Most of the time | 3. All of the time |
|  | 5-Private sector role in health sector policy, supply chain and service delivery is promoted by law and regulated by different regulatory bodies | 0.None of the time | 1. Some of the time | 2. Most of the time | 3. All of the time |
|  | ***Please select the response code which expresses your best opinion about the following statements. If you do not know or think that that statement is not applicable, then please use zero response category. In ACCOUNTABLE healthcare*** | | | | |
| **● Mechanisms are in place to ensure patients’ privacy and satisfaction with care;** | 1 - Clients' privacy is maintained during consultation with health provider | 0.None of the time | 1. Some of the time | 2. Most of the time | 3. All of the time |
|  | 2 - Clients are treated with respect | 0.None of the time | 1. Some of the time | 2. Most of the time | 3. All of the time |
|  | 3 - Clients are involved in their care decision-making | 0.None of the time | 1. Some of the time | 2. Most of the time | 3. All of the time |
|  | 4 - Clients can choose their health providers | 0.None of the time | 1. Some of the time | 2. Most of the time | 3. All of the time |
|  | 5 – Health staff give preferential treatment to family, friends, and people in position of authority | 0.None of the time | 1. Some of the time | 2. Most of the time | 3. All of the time |
|  | ***Please select the response code which expresses your best opinion about the following statements. If you do not know or think that that statement is not applicable, then please use zero response category. In ACCOUNTABLE healthcare*** | | | | |
| **● Information regarding financing, delivery and outcomes of care (at a population level) is publicly available;** | 1-Existing routine information systems tend to inflate data on service coverage and health system functions (human resource, financing, logistics, etc.) | 0.None of the time | 1. Some of the time | 2. Most of the time | 3. All of the time |
|  | 2- Actions are implemented to integrate routine data from different information systems through interoperable information architecture | 0.None of the time | 1. Some of the time | 2. Most of the time | 3. All of the time |
|  | 3- Data quality is checked to maintain high level of accuracy | 0.None of the time | 1. Some of the time | 2. Most of the time | 3. All of the time |
|  | 4- Organizational processes are functional to foster use of data at different levels | 0.None of the time | 1. Some of the time | 2. Most of the time | 3. All of the time |
|  | 5- Mechanisms are active to make health data publicly available for accountability and benchmarking | 0.None of the time | 1. Some of the time | 2. Most of the time | 3. All of the time |
|  | ***Please select the response code which expresses your best opinion about the following statements. If you do not know or think that that statement is not applicable, then please use zero response category. In ACCOUNTABLE healthcare*** | | | | |
| **● Good health outcomes, supported by self-directed and family-centered positive health behaviors, are sustained;** | 1-Patients are inquired and counselled about their health behaviors | 0.None of the time | 1. Some of the time | 2. Most of the time | 3. All of the time |
|  | 2-Patients are reminded of their behavior/medication compliance and health provider appointments | 0.None of the time | 1. Some of the time | 2. Most of the time | 3. All of the time |
|  | 3-Patients are tracked for continuity of care and for better outcomes | 0.None of the time | 1. Some of the time | 2. Most of the time | 3. All of the time |
|  | 4-Health providers are not rewarded for positive changes in healthy behaviors in their catchment area populations | 0.None of the time | 1. Some of the time | 2. Most of the time | 3. All of the time |
|  | 5-Health providers are rewarded for positive changes in health behaviors and outcomes in their catchment area populations | 0.None of the time | 1. Some of the time | 2. Most of the time | 3. All of the time |
|  | ***Please select the response code which expresses your best opinion about the following statements. If you do not know or think that that statement is not applicable, then please use zero response category. In ACCOUNTABLE healthcare*** | | | | |
| **● Licensing agencies and professional organizations are responsible for credentialing providers, accrediting facilities, and setting standards in partnership with national (and/or local) government** | 1-The health training institutions quality is regulated by professional licensing bodies | 0.None of the time | 1. Some of the time | 2. Most of the time | 3. All of the time |
|  | 2-The health providers quality is regulated by professional councils/bodies | 0.None of the time | 1. Some of the time | 2. Most of the time | 3. All of the time |
|  | 3-The continuing education is administered for all types of health providers | 0.None of the time | 1. Some of the time | 2. Most of the time | 3. All of the time |
|  | 4- People believe that the health facilities accreditation process is compromised | 0.None of the time | 1. Some of the time | 2. Most of the time | 3. All of the time |
|  | 5-The facilities employ quality improvement methods to solve problems and improve performance | 0.None of the time | 1. Some of the time | 2. Most of the time | 3. All of the time |
|  | ***Please select the response code which expresses your best opinion about the following statements. If you do not know or think that that statement is not applicable, then please use zero response category. In ACCOUNTABLE healthcare*** | | | | |
| **● Recourse or appeal options are available for patients or communities dissatisfied with health care.** | 1-Mechanisms are functional for clients to directly report their complaints to district or higher level | 0.None of the time | 1. Some of the time | 2. Most of the time | 3. All of the time |
|  | 2-The facilities/districts provide feedback and address clients complaints within 72 hours | 0.None of the time | 1. Some of the time | 2. Most of the time | 3. All of the time |
|  | 3- Facility and district management committees comprising of staff and community members/leaders exist to address clients’ complaints | 0.None of the time | 1. Some of the time | 2. Most of the time | 3. All of the time |
|  | 4- People believe that health staff do not accept their mistakes and do not use those mistakes to learn and improve | 0.None of the time | 1. Some of the time | 2. Most of the time | 3. All of the time |
|  | 5-District and higher level leadership monitor clients' complaints through telephone text message or through other mechanisms and respond to grievance | 0.None of the time | 1. Some of the time | 2. Most of the time | 3. All of the time |

| **In Accessible Health Care** | **Indicators**  ***Please select the response code which expresses your best opinion about the following statements. If you do not know or think that that statement is not applicable, then please use zero response category. In ACCESSIBLE healthcare*** | | | | |
| --- | --- | --- | --- | --- | --- |
| **● Health facilities and medicines are located within a reasonable distance, are consistently open on a regular schedule known to the community, have staff and equipment to fulfill their designated functions;** | 1 - Health facilities providing essential services are located within 5 miles or 8 km radius of where people reside (village, town, city) | 0.None of the time | 1. Some of the time | 2. Most of the time | 3. All of the time |
|  | 2 - Health facilities have all required staff and equipment as per the standards for different types of facilities | 0.None of the time | 1. Some of the time | 2. Most of the time | 3. All of the time |
|  | 3 - Pharmacy is located within two miles/3 km or 15 minutes travel time from the health facility | 0.None of the time | 1. Some of the time | 2. Most of the time | 3. All of the time |
|  | 4 - Mechanisms are functional to identify poor, vulnerables, and marginalized to improve access to services | 0.None of the time | 1. Some of the time | 2. Most of the time | 3. All of the time |
|  | 5 - Health facilities open for eight hours but have someone on call after hours to provide emergency care | 0.None of the time | 1. Some of the time | 2. Most of the time | 3. All of the time |
|  | ***Please select the response code which expresses your best opinion about the following statements. If you do not know or think that that statement is not applicable, then please use zero response category. In ACCESSIBLE healthcare*** | | | | |
| **● Alternative care options exist to extend the reach of traditional health facilities, including both paid and voluntary community health workers, as well as digital or e-health applications, drug shops/pharmacies, mobile outreach, etc.;** | 1 - Community based health workers provide preventive services, referral and distribute contraceptive commodities, ORS, malaria, TB, and HIV/AIDS medicines as per country policy | 0.None of the time | 1. Some of the time | 2. Most of the time | 3. All of the time |
|  | 2 – Health facility conduct outreach activities for mobilizing communities for utilizing MNCH, FP, Malaria, TB, HIV/AIDS and other essential services | 0.None of the time | 1. Some of the time | 2. Most of the time | 3. All of the time |
|  | 3 - Mobile health services are available for remote and dispersed populations | 0.None of the time | 1. Some of the time | 2. Most of the time | 3. All of the time |
|  | 4 - Digital health applications available for to facilitate community workers services | 0.None of the time | 1. Some of the time | 2. Most of the time | 3. All of the time |
|  | 5 - Telemedicine exist to support nurses/doctors in remote areas | 0.None of the time | 1. Some of the time | 2. Most of the time | 3. All of the time |
|  | ***Please select the response code which expresses your best opinion about the following statements. If you do not know or think that that statement is not applicable, then please use zero response category. In ACCESSIBLE healthcare*** | | | | |
| **● Quality of care for priority interventions meets established standards from both providers and consumers, and is consistent across all facilities, regardless of whether they are public, private, non-profit, or faith-based;** | 1 – Quality of care policies implemented both in public and private sectors | 0.None of the time | 1. Some of the time | 2. Most of the time | 3. All of the time |
|  | 2 – Compliance with quality of care standards for priority health services is monitored through facility self-assessment or by district or higher level | 0.None of the time | 1. Some of the time | 2. Most of the time | 3. All of the time |
|  | 3 - Quality improvement is used for dealing with processes of care to improve performance at all levels | 0.None of the time | 1. Some of the time | 2. Most of the time | 3. All of the time |
|  | 4 - Mechanisms are operational to educate public to demand and advocate for quality of MNCH, FP, Malaria, TB, HIV/AIDS and other essential services | 0.None of the time | 1. Some of the time | 2. Most of the time | 3. All of the time |
|  | 5 - Mechanisms are operational to assess public awareness about detecting quality of medicines, vaccines and medical technologies | 0.None of the time | 1. Some of the time | 2. Most of the time | 3. All of the time |
|  | ***Please select the response code which expresses your best opinion about the following statements. If you do not know or think that that statement is not applicable, then please use zero response category. In ACCESSIBLE healthcare*** | | | | |
| **● Emergency health care and related transportation are available.** | 1 - Access to emergency obstetric care available within 5 miles or 8 Km radius of where people reside (village, town, city) | 0.None of the time | 1. Some of the time | 2. Most of the time | 3. All of the time |
|  | 2 - Access to domestic violence trauma services available within 5 mile radius where population reside (village, town, city) | 0.None of the time | 1. Some of the time | 2. Most of the time | 3. All of the time |
|  | 3 - Health facility has functional ambulance to provide transport to access emergency care when needed and transfer patients/clients to hospitals | 0.None of the time | 1. Some of the time | 2. Most of the time | 3. All of the time |
|  | 4 - Communities have a transport system to transfer patients/clients to the nearby facility | 0.None of the time | 1. Some of the time | 2. Most of the time | 3. All of the time |
|  | 5 - All physicians deployed in rural and remote areas are trained in caesarian section | 0.None of the time | 1. Some of the time | 2. Most of the time | 3. All of the time |
|  | ***Please select the response code which expresses your best opinion about the following statements. If you do not know or think that that statement is not applicable, then please use zero response category. In ACCESSIBLE healthcare*** | | | | |
| **● People understand when, why, and where to get the care they need and are motivated to seek it.** | 1 – Health providers, outreach workers and CHWs educate people about available health facilities and MNCH, FP, Malaria, TB, HIV/AIDS and other essential services | 0.None of the time | 1. Some of the time | 2. Most of the time | 3. All of the time |
|  | 2 – Health provider, outreach workers and CHWs educate people about priority health issues and when to seek care | 0.None of the time | 1. Some of the time | 2. Most of the time | 3. All of the time |
|  | 3 – Actions are taken to encourage people and leaders adopt new health practices and lifestyle to promote health | 0.None of the time | 1. Some of the time | 2. Most of the time | 3. All of the time |
|  | 4 – Community mobilization activities are conducted to increase peoples’ participation in health needs advocacy and for monitoring health outcomes | 0.None of the time | 1. Some of the time | 2. Most of the time | 3. All of the time |
|  | 5 – Information from different sources is triangulated to track increase in MNCH, FP, Malaria, TB, HIV/AIDS service utilization | 0.None of the time | 1. Some of the time | 2. Most of the time | 3. All of the time |
|  | ***Please select the response code which expresses your best opinion about the following statements. If you do not know or think that that statement is not applicable, then please use zero response category. In ACCESSIBLE healthcare*** | | | | |
| **● Providers deliver health care in a manner that ensures equitable health outcomes and promotes dignity and respect for all patients and providers.** | 1 - Health facility staff treat everyone equally irrespective of their economic status, religion, ethnicity, race, caste, creed, etc. | 0.None of the time | 1. Some of the time | 2. Most of the time | 3. All of the time |
|  | 2 - Health staff promote women autonomy to negotiate and make her own health decisions | 0.None of the time | 1. Some of the time | 2. Most of the time | 3. All of the time |
|  | 3 – Health providers ensure representations in facility management committee from different social groups, gender and poor for improving health equity among these groups | 0.None of the time | 1. Some of the time | 2. Most of the time | 3. All of the time |
|  | 4 – Facility and district staff disaggregate MNCH, FP, Malaria, TB, HIV/AIDS services utilization data by gender, geography and income and other vulnerable groups to track reduction in health outcomes disparities | 0.None of the time | 1. Some of the time | 2. Most of the time | 3. All of the time |
|  | 5 -Mechanisms are in place to improve gender balance in health workforce to improve access to female providers and choice of health providers | 0.None of the time | 1. Some of the time | 2. Most of the time | 3. All of the time |

| **In Affordable Health Care** | **Indicators**  ***Please select the response code which expresses your best opinion about the following statements. If you do not know or think that that statement is not applicable, then please use zero response category. In AFFORDABLE healthcare*** | | | | |
| --- | --- | --- | --- | --- | --- |
| **● Routine or unexpected health care costs, including medications or supplies do not impoverish people;** | 1 - The public health facilities provide health care to everyone irrespective of their paying capacity | 0.None of the time | 1. Some of the time | 2. Most of the time | 3. All of the time |
|  | 2 - Private health providers charge less to poor people | 0.None of the time | 1. Some of the time | 2. Most of the time | 3. All of the time |
|  | 3 - Hospitals (public/private) have charity funds to treat people who could not bear costs of health care | 0.None of the time | 1. Some of the time | 2. Most of the time | 3. All of the time |
|  | 4 - Emergency care is free in public hospitals in principle, but people are ask to pay for medicines/supplies | 0.None of the time | 1. Some of the time | 2. Most of the time | 3. All of the time |
|  | 5 - People believe that the government social protection schemes for poor and vulnerable do not strictly follow eligibility criteria | 0.None of the time | 1. Some of the time | 2. Most of the time | 3. All of the time |
|  | ***Please select the response code which expresses your best opinion about the following statements. If you do not know or think that that statement is not applicable, then please use zero response category. In AFFORDABLE healthcare*** | | | | |
| **● People continue to seek needed care after considering the total cost of that care (e.g., cost of services, drugs, supplies, transport and care for family members left behind or who accompany the patient);** | 1 - The costs of medical consultation at public health facilities including hospital is: | 0.None | 1. Low | 2. Medium | 3. High |
|  | 2 - The costs of medical consultation at private health facilities is: | 0.None | 1. Low | 2. Medium | 3. High |
|  | 3 - The costs of specialized treatment and hospitalization both in public and private hospitals are: | 0.None | 1. Low | 2. Medium | 3. High |
|  | 4 - The costs of medicines and supplies are: | 0.None | 1. Low | 2. Medium | 3. High |
|  | 5 - The costs of other tests, x-rays, supplies are: | 0.None | 1. Low | 2. Medium | 3. High |
|  | ***Please select the response code which expresses your best opinion about the following statements. If you do not know or think that that statement is not applicable, then please use zero response category. In AFFORDABLE healthcare*** | | | | |
| **● People opt to participate in pre-payment schemes or insurance plans to improve their ability to access health care and be protected from financial hardship due to illness;** | 1 - Employers are required to provide health insurance with some premium paid by employee | 0.None of the time | 1. Some of the time | 2. Most of the time | 3. All of the time |
|  | 2 - Industrial workers are insured under social security schemes | 0.None of the time | 1. Some of the time | 2. Most of the time | 3. All of the time |
|  | 3 - Private insurance is available for those who want to buy it | 0.None of the time | 1. Some of the time | 2. Most of the time | 3. All of the time |
|  | 4 - Social protection schemes exist to cover all types of health costs for poor/near poor, old and disabled people as defined by the government | 0.None of the time | 1. Some of the time | 2. Most of the time | 3. All of the time |
|  | 5 - National health insurance scheme covers everyone along with private option | 0.None of the time | 1. Some of the time | 2. Most of the time | 3. All of the time |
|  | ***Please select the response code which expresses your best opinion about the following statements. If you do not know or think that that statement is not applicable, then please use zero response category. In AFFORDABLE healthcare*** | | | | |
| **● Governments allocate financial and human resources for health to meet priority needs, and work with the private sector and civil society to increase domestic funding and ensure adequate distribution of such resources** | 1-Government health funding is directed towards priority needs | 0.No | 1.<25% | 2.50-75% | 3.>75% |
|  | 2-Government health funding is directed towards its Military and civil service staff | 0.No | 1.<25% | 2.50-75% | 3.>75% |
|  | 3-Government health funding subsidize health cost for poor and vulnerable | 0.No | 1.<25% | 2.50-75% | 3.>75% |
|  | 4-Government health allocation is boosted by private sector, civil society | 0.No | 1.<25% | 2.50-75% | 3.>75% |
|  | 5-Government health funding is supplemented by donors | 0.No | 1.<25% | 2.50-75% | 3.>75% |
|  | ***Please select the response code which expresses your best opinion about the following statements. If you do not know or think that that statement is not applicable, then please use zero response category. In AFFORDABLE healthcare*** | | | | |
| **● Safe and effective essential medicines are available without undue financial hardship to obtain them, along with mechanisms to ensure their responsible use ( at the right time, for the right conditions, and at the right doses).** | 1 - Mechanisms are functional to negotiate medicines prices in the country and make it affordable to general public | 0.None of the time | 1. Some of the time | 2. Most of the time | 3. All of the time |
|  | 2 - Mechanisms/schemes are operational to pay for expensive medicines for certain conditions for poor patients in public and private sector | 0.None of the time | 1. Some of the time | 2. Most of the time | 3. All of the time |
|  | 3 - Monitoring rationale use of medicine, adverse reactions and safe disposal of expired medicines is ineffective | 0.None of the time | 1. Some of the time | 2. Most of the time | 3. All of the time |
|  | 4 - Federal/national drug administration or other regulatory bodies are functional to ensure affordable quality medicines and contraceptive commodities in the market place | 0.None of the time | 1. Some of the time | 2. Most of the time | 3. All of the time |
|  | 5 - Supply chain maintenance costs are reasonable to ensure timely availability of safe and quality medicines and contraceptive commodities | 0.None of the time | 1. Some of the time | 2. Most of the time | 3. All of the time |

| **In Reliable Health Care** | **Indicators**  ***Please select the response code which expresses your best opinion about the following statements. If you do not know or think that that statement is not applicable, then please use zero response category. In RELIABLE healthcare*** | | | | |
| --- | --- | --- | --- | --- | --- |
| **● Health facilities and health workers have the right supplies and quantity of commodities needed to deliver care, including running water and sanitation services, reliable sources of energy, and appropriate procedures to prevent infections;** | 1 – Districts or higher level authorities plan and deploy workforce in time, according to facility workforce standards | 0.None of the time | 1. Some of the time | 2. Most of the time | 3. All of the time |
|  | 2 - Districts or higher level authorities plan and deliver required supplies/commodities (types and quantities) on time in rural areas | 0.None of the time | 1. Some of the time | 2. Most of the time | 3. All of the time |
|  | 3 - All facilities in rural areas have reliable supply of electricity, running water and sanitation service | 0.None of the time | 1. Some of the time | 2. Most of the time | 3. All of the time |
|  | 4 – People believe that health facilities inflate or over-report consistent implementation of infection prevention/control protocol | 0.None of the time | 1. Some of the time | 2. Most of the time | 3. All of the time |
|  | 5 - Disruption in supplies, commodities, electricity, running water, sanitation and infection control are tracked and addressed within 72 hours | 0.None of the time | 1. Some of the time | 2. Most of the time | 3. All of the time |
|  | ***Please select the response code which expresses your best opinion about the following statements. If you do not know or think that that statement is not applicable, then please use zero response category. In RELIABLE healthcare*** | | | | |
| **● Health workers have the knowledge, skills, motivation, credentials and cultural understanding to provide care; are engaged in continuing education; and are regulated through professional and licensing associations, and supported through retention strategies.** | 1 - Health providers are well trained and provide quality services | 0.None of the time | 1. Some of the time | 2. Most of the time | 3. All of the time |
|  | 2 - Health providers are well trained to provide care without discrimination and stigma | 0.None of the time | 1. Some of the time | 2. Most of the time | 3. All of the time |
|  | 3 - Health providers trust regulatory bodies high level of professionalism | 0.None of the time | 1. Some of the time | 2. Most of the time | 3. All of the time |
|  | 4 – To increase retention, health workers are appreciated and supported for their work and professional growth | 0.None of the time | 1. Some of the time | 2. Most of the time | 3. All of the time |
|  | 5 - Continuing education is available for all types of health providers for keeping them updated with latest knowledge and skills | 0.None of the time | 1. Some of the time | 2. Most of the time | 3. All of the time |
|  | ***Please select the response code which expresses your best opinion about the following statements. If you do not know or think that that statement is not applicable, then please use zero response category. In RELIABLE healthcare*** | | | | |
| **● Health facilities are able to meet the standards of accrediting organizations, effectively engage their communities and the people they serve;** | 1 - Health training institutions meet accreditation requirements of the regulatory bodies | 0.None of the time | 1. Some of the time | 2. Most of the time | 3. All of the time |
|  | 2 – Health providers believe that the facility accreditation process is transparent | 0.None of the time | 1. Some of the time | 2. Most of the time | 3. All of the time |
|  | 3 – Mechanisms are in place to support public facilities in meeting accreditation standards | 0.None of the time | 1. Some of the time | 2. Most of the time | 3. All of the time |
|  | 4 - Community engagement through community leaders or civil society is one of the criteria for facility accreditation | 0.None of the time | 1. Some of the time | 2. Most of the time | 3. All of the time |
|  | 5 - Facility accreditation scores are publicly available for accountability and benchmarking | 0.None of the time | 1. Some of the time | 2. Most of the time | 3. All of the time |
|  | ***Please select the response code which expresses your best opinion about the following statements. If you do not know or think that that statement is not applicable, then please use zero response category. In RELIABLE healthcare*** | | | | |
| **● Systems to manage pharmaceutical and logistics are in place so that medicines, devices and commodities are safe and of expected quality, and with controls that minimize the risks of theft or falsification;** | 1 - Food, medical technology and drug regulatory authorities are fully functional | 0.None of the time | 1. Some of the time | 2. Most of the time | 3. All of the time |
|  | 2 - Mechanisms are functional to check quality of medicines and contraceptive commodities produced in the country | 0.None of the time | 1. Some of the time | 2. Most of the time | 3. All of the time |
|  | 3 – Regulatory agency register, check and regulate quality of imported medicines | 0.None of the time | 1. Some of the time | 2. Most of the time | 3. All of the time |
|  | 4 – Monitoring mechanisms report regularly on safe disposal of discarded medicines and contraceptive commodities | 0.None of the time | 1. Some of the time | 2. Most of the time | 3. All of the time |
|  | 5 - Mechanisms are in place to track delivery of medicines from storage to health facilities and pharmacies | 0.None of the time | 1. Some of the time | 2. Most of the time | 3. All of the time |
|  | ***Please select the response code which expresses your best opinion about the following statements. If you do not know or think that that statement is not applicable, then please use zero response category. In RELIABLE healthcare*** | | | | |
| **● Health workers are safe from violence, assault, or harassment and are protected against natural disasters, disease outbreaks and emergencies;** | 1 - Government provide safety protection when needed such as threat from terrorists, criminal, etc. | 0.None of the time | 1. Some of the time | 2. Most of the time | 3. All of the time |
|  | 2 - Health staff are trained regularly on sexual harassment reporting, prevention, and remedy | 0.None of the time | 1. Some of the time | 2. Most of the time | 3. All of the time |
|  | 3 – Health facilities have consistent supply of materials and equipment for infection control | 0.None of the time | 1. Some of the time | 2. Most of the time | 3. All of the time |
|  | 4 - Health facilities are connected to animal health surveillance and early warning system to handle natural and man-made disasters | 0.None of the time | 1. Some of the time | 2. Most of the time | 3. All of the time |
|  | 5 - Health staff are regularly trained on emergency protocol, communication and coordination with communities, district, other facilities, first responders, law enforcements, higher authorities, etc. | 0.None of the time | 1. Some of the time | 2. Most of the time | 3. All of the time |
|  | ***Please select the response code which expresses your best opinion about the following statements. If you do not know or think that that statement is not applicable, then please use zero response category. In RELIABLE healthcare*** | | | | |
| **● Patients trust that institutions and providers will give them the care they need in a way that meets their needs respectfully without stigma, shame, fear or abuse** | 1 - People **trust** health providers to provide care they need or refer them to specialist | 0.No | 1. Low | 2. Medium | 3. High |
|  | 2 - People have **confidence** in their health providers to share their confidential medical records | 0.No | 1. Low | 2. Medium | 3. High |
|  | 3 – People **expect** from health providers will identify intimate partner or domestic violence and sexual abuse | 0.No | 1. Low | 2. Medium | 3. High |
|  | 4 - People have **hope** that health providers will work with law enforcement in confidence to protect clients from violence and abuse | 0.No | 1. Low | 2. Medium | 3. High |
|  | 5 - People **assume** that the health providers will educate people in reducing stigma attached to certain diseases and domestic violence | 0.No | 1. Low | 2. Medium | 3. High |
|  | ***Please select the response code which expresses your best opinion about the following statements. If you do not know or think that that statement is not applicable, then please use zero response category. In RELIABLE healthcare*** | | | | |
| **● Care continues during times of disruption, shock, or crisis.** | 1 - Health facilities have processes in place for task-shifting and task sharing in times of crisis | 0.None of the time | 1. Some of the time | 2. Most of the time | 3. All of the time |
|  | 2 - Health facilities have processes for bringing in additional staff in times of crisis | 0.None of the time | 1. Some of the time | 2. Most of the time | 3. All of the time |
|  | 3 - Health facilities have extra medicines, contraceptive commodities and supplies to meet any crisis needs | 0.None of the time | 1. Some of the time | 2. Most of the time | 3. All of the time |
|  | 4 - Health facilities have alternative energy sources to continue operations in times of crisis | 0.None of the time | 1. Some of the time | 2. Most of the time | 3. All of the time |
|  | 5 - Health facilities have coordinating mechanisms with first responders and law enforcement officials to keep peace and avoid disruption in services | 0.None of the time | 1. Some of the time | 2. Most of the time | 3. All of the time |

**Section Six - Other**

| **Please select response code which expresses your best opinion about the statement.** | | | | |
| --- | --- | --- | --- | --- |
| 1 - In your estimate, what percentage of pregnant women have access to skilled birth attendant? | 1. Less than 25% | 2. 25-49 % | 3. 50-74% | 4.75% or more |
| 2 - In your estimate, what percentage of children are fully immunized? | 1. Less than 25% | 2. 25-49 % | 3. 50-74% | 4.75% or more |
| 3 - In your estimate, how much people pay out of pocket of the total cost of their health visit when they are sick? | 1. Less than 25% | 2. 25-49 % | 3. 50-74% | 4.75% or more |
| 4 - In your estimate, how many government facilities meet staffing standards requirements? | 1. Less than 25% | 2. 25-49 % | 3. 50-74% | 4.75% or more |
| 5 - In your estimate, what is the average international health regulation (IHR) capacity score for your country? | 1. Less than 25% | 2. 25-49 % | 3. 50-74% | 4.75% or more |
|  | | | | |
| **Section 7 -Please provide your comments.** | | | | |
| **Please provide your comments and suggestions. If you think that some statements are difficult to understand and need revision, please describe them, or note their number. Also note which statements are not relevant. If space is limited in first box, use the second box of comments and suggestion** | | | | |
| **Comments and suggestions** | | | | |
| **Comments and suggestions** | | | | |
